# Parasite Replication and the Evolutionary Epidemiology of Parasite Virulence

**DOI:** 10.1371/journal.pone.0012440

**Published:** 2010-08-27

**Authors:** Michael B. Bonsall

**Affiliations:** 1 Mathematical Ecology Research Group, Department of Zoology, University of Oxford, Oxford, United Kingdom; 2 St. Peter's College, Oxford, United Kingdom; University of Swansea, United Kingdom

## Abstract

Parasite virulence evolution is shaped by both within-host and population-level processes yet the link between these differing scales of infection is often neglected. Population structure and heterogeneity in both parasites and hosts will affect how hosts are exploited by pathogens and the intensity of infection. Here, it is shown how the degree of relatedness among parasites together with epidemiological parameters such as pathogen yield and longevity influence the evolution of virulence. Furthermore, the role of kin competition and the degree of cheating within highly structured parasite populations also influences parasite fitness and infectivity patterns. Understanding how the effects of within-host processes scale up to affect the epidemiology has importance for understanding host-pathogen interactions.

## Introduction

Virulence, the pathogen-induced reduction in host fitness (through either increased host mortality or reduced host growth rate) is an emergent epidemiological property of a parasite through its interaction with its host. Alternatively, virulence is defined as the ability of a parasite to overcome a given level of host resistance. This concept has given rise to co-evolutionary genetic (e.g. gene-for-gene) mechanisms for the evolution of virulence (particularly in plant-parasite interactions [1]). However, the traditional view that evolutionary change in parasite virulence leads to a reduction in the capacity to induce harm to hosts and prolong host longevity favouring parasite fitness has been challenged on many occasions and in many different studies [2]–[7]. Parasite virulence is a complex product of many interactions within a host and can depend on a range of different factors that are responsible for the expression of the trait. These include the sublethal expression of protein toxins [8], [9] that affect the life history characteristics of the host [10], the life history of the parasite [6], [11]–[12], the multiplicity of infections [13], [14] and temporal and/or spatial environmental heterogeneity [15]–[18].

Although parasite virulence is explicitly integrated to within-host processes, the evolution of this parasite trait may not necessarily be linked to other parasite life history strategies operating at different scales such as pathogen transmission between hosts [19], [20]. Virulence can act in a local, narrow way and lead to high-levels of morbidity (or mortality) within an individual host such that optimal transmission at an epidemiological level might be compromised [21]. The structure of the microenvironment of individual hosts [22], interactions amongst parasites through competition and cooperation [23]–[25] and the effects of the host immune system [7], [26]–[27] can all contribute to the differential evolution of virulence amongst hosts. Broadly, heterogeneities will affect virulence evolution [16] as patterns of parasite replication alter host exploitation and affect the intensity of infection.

This heterogeneity and patterns of host exploitation will affect the genetic structure of pathogen populations. Under low levels of pathogen recombination [28], parasite replication within hosts allows population structure to form [29]. This population-level viscosity is known to affect the degree of relatedness and the ecology of local interactions [30]–[34]. While viscosity affects inclusive fitness by increasing the degree of relatedness [30]–[31] local competition between kin can decrease any benefits to social interactions [32]–[34]. More recently, and in the context of host-pathogen interactions, the role of local interactions and competition has been shown to affect patterns of pathogen infectivity [18] and epidemiology (see [35] for a review).

While the epidemiology of host-pathogen interactions is well established [36], [37] the effect of coupling the within-host and epidemiological consequences of parasitic infections is a relatively novel advance in parasite evolutionary ecology [35]. Here, the goal of this study is to address this issue and explore how patterns of parasite replication affect the broader epidemiological consequences of host-pathogen interactions. In explicitly considering this link it becomes essential to separate out the effects of competition from the effects of replication on the evolution of parasite life history traits. For instance, the effects of kin competition and parasite replication can be considered as two mechanisms by which cooperation and collective actions might be manifest. Reducing kin competition can be viewed as a cooperative behaviour and replication within a host allows successful resource exploitation (often through the production of shared public goods).

As outlined, the aim of this study is to explore, theoretically, how within-host pathogen replication affects parasite fitness and epidemiology. After introducing a description of pathogen fitness involving replicating and non-replicating strategies, the optimal replication strategy together with the effects of relatedness on parasite fitness is derived. Following on from this, the epidemiological consequences of within-host replication are explored in a host-pathogen model in which the pathogen is an obligate killer and has free-living infectious stages. These results are discussed with reference to recent developments in host-pathogen epidemiology and virulence evolution.

## Within-Host Dynamics

### Parasite Replication

Parasite fitness is assumed to be dependent on the parasite's own replication strategy and on the effects of the average replication strategy across the group of parasites within a host. In more detail, the fitness function can be derived in terms of whether a parasite adopts to undergo costly replication or is a strategy that chooses not to replicate to kill the host but utilizes resources from the dead cadaver. The relative success of a parasite that replicates is a function of the costs of replication, the strength of competition from these related (kin) parasites and the proportion of non-kin in the host. This biology is described by:
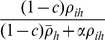
(1)where *ρ_ih_* is the replication fraction of the *i^th^* parasite in the *h^th^* host, 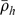 is the average replication fraction across the group of parasites in a host, *c* is the costs associated with replicating, *α* is the strength of competition. Thus 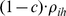 is the fitness benefit to parasites that replicate relative to the strength of competition amongst related parasites (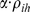) and the strength of interactions with replicating non-kin (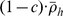).

Importantly, the strength of competition (α) amongst the focal parasite strain is the key feature of the fitness function and determines the relative strength of the interaction amongst kin and consequently pathogen growth and abundance within a host. If α>1 kin compete more strongly amongst themselves, whereas if α<1 kin compete less intensely amongst themselves and, therefore, may act cooperatively.

**Figure 1:**
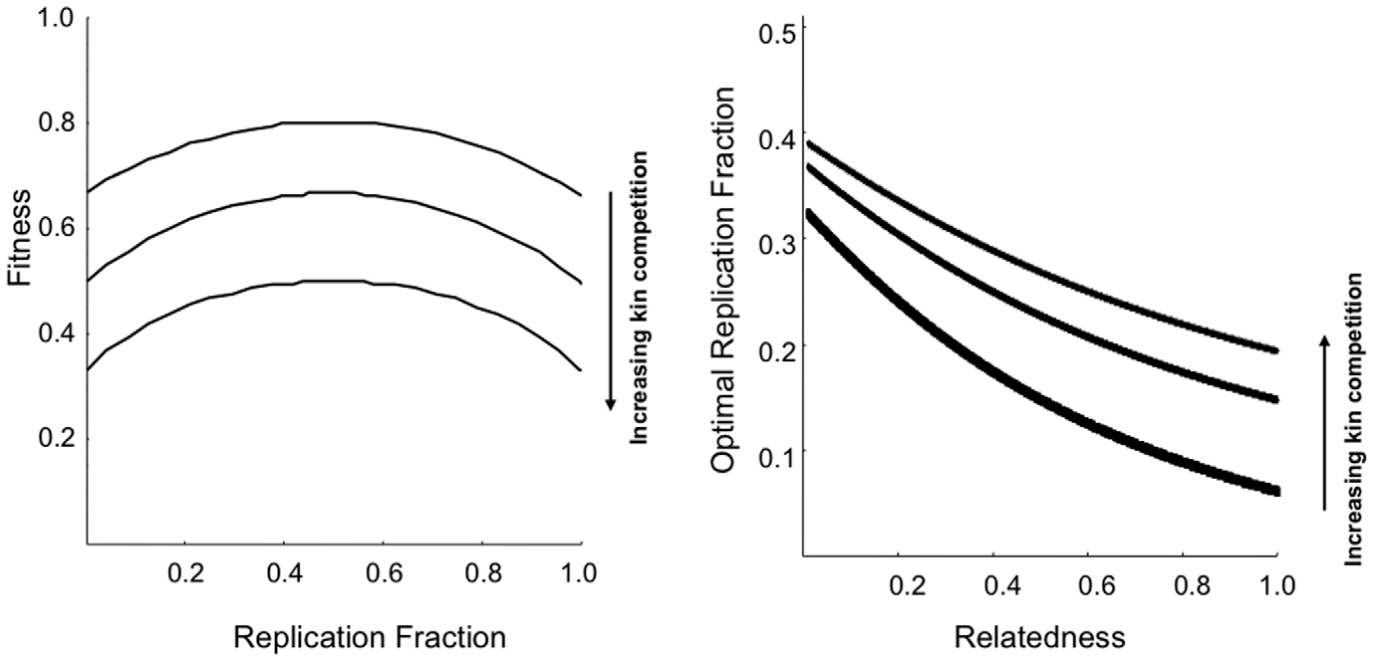
*The role of parasite replication and relatedness on fitness.* (A) Parasite fitness is a non-linear function of replication fraction (*ρ_ih_*) and declines with increasing kin competition (*α*). (B) The optimal replication fraction declines with increasing levels of relatedness (*R*) within a host as prudent exploitation strategies predominant and increases with increasing kin competition (Other parameters *η* = 1, *c* = 0.5, 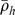 = 0.5).

The impact of non-replicating parasites 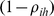 on fitness can be determined from the proportion of non-replicators relative to the strength of competition amongst non-replicators and the presence of non-kin. This biology is captured with:
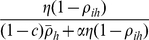
(2)where 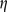 is the strength of non-replicating parasites (0<
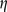
<1) relative to the replicating strategy. Non replicating parasites do not any pay costs associated with growth but may affect overall host morbidity and mortality (see below). Given all of this, the overall fitness (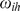) of the *i^th^* parasite phenotype in the *h^th^* host can be defined as:
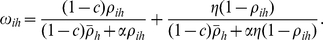
(3)Parasite fitness is a non-linear function of replication strategy (**Figure 1a**). Low replication strategies have low fitness as parasite growth rate is restricted. Similarly high levels of replication have low fitness as the costs of replication act to restrict fitness. Increases in the strength of kin competition (*α*) also act to decrease fitness (**Figure 1a**).

The neighbour-modulated (inclusive fitness) effects of replicating and non-replicating parasites [38] can be determined by considering how an alternative parasite strategy (with a different replication phenotype: 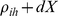) alters fitness [39]. In doing so it becomes necessary to evaluate how a parasite alters both direct and indirect fitness under weak selection. This is done through a fitness maximisation method using the multivariate chain rule [39], [40]:
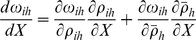
(4)where the ratio 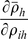 is a statistical measure of relatedness [41] (and is the change in the average phenotype with respect to changes in the focal phenotype – [39]).

The optimal replication fraction is found by setting equation 4 equal to zero and solving for 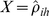, which in the absence of the non-replicator strategy (*η* = 0), yields
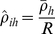
(5)where *R* is the degree of relatedness. The optimal replication strategy declines with increasing relatedness. The presence of non-replicators affects the optimal parasite strategy and as the strength of non-replicators increases (*η* = 1), the optimal replication fraction (under minimal replication costs, *c*→0) is then the positive solution from:
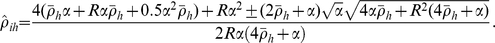
(6)Under different levels of kin competition the optimal replication fraction declines as relatedness increases (**Figure 1b**). In unrelated environments (*R*→0), the optimal replication strategy is high leading to high host exploitation. When parasites are related (*R*→1), the optimal replication strategy is low favouring prudent host exploitation (**Figure 1b**). Reduced kin competition (α<1) gives rise to lower optimal strategies as cooperative actions are more prevalent and leads to the prudent exploitation of the host by the parasite. Increasing the effects of the non-replicator effects (increasing *η*) reduces the prudent exploitation strategy and facilitates higher optimal replication strategies leading to high host exploitation.

### Relatedness

Relatedness is not a fixed quantity but a statistical relationship dependent on parasite density [41]. As such, the degree of relatedness (*R*) amongst (haploid) parasites within a host can be determined from standard population genetic theory [42]:
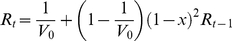
(7)where *V_0_* is the effective parasite population size and *x* is the mutation rate. At equilibrium (R^*^),
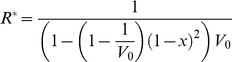
(8)Together with equation (6), this expression (equation 8) allows the optimal parasite replication strategy to be expressed in terms of the degree of relatedness and the effective parasite population size (within a host). Effective population size is driven by the strength of competition amongst parasites within a host (α) and hence understanding the within- and between-host epidemiological processes is critical in order to determine an appropriate measure of pathogen fitness.

## Host-Pathogen Dynamics

In order to link the within-host replication dynamics to the epidemiology of host-pathogen interaction a host-pathogen epidemiological framework (e.g., [36]) is developed in which the pathogen (*V*) is lethal to the host (*H*) and has free-living infectious stages. Following infection, infected hosts (*I*) die and cadavers yield a number of free-living pathogen particles. Pathogen replication (*ρ*) directly affects pathogen virulence (*m*(*ρ*)), and indirectly affects pathogen yield (through virulence) (*f*(*m*)). The epidemiological model is of the form:
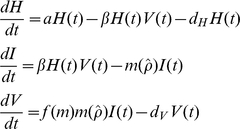
(9–11)where *a* is the host birth rate, *ρ* is the pathogen transmission rate, *d_H_* is the host death rate (independent of both host and parasite density) and *d_V_* is the death rate of the free-living pathogen. Virulence, the pathogen induced mortality rate (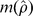) is assumed to be a decelerating (convex) function of within-host replication fraction such that 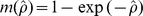 and pathogen yield (*f*(*m*)) following death of the host is an increasing linear function of pathogen replication rate such that 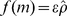, where *ε* is a positive scaling constant linking virulence and yield (yield strength).

**Figure 2:**
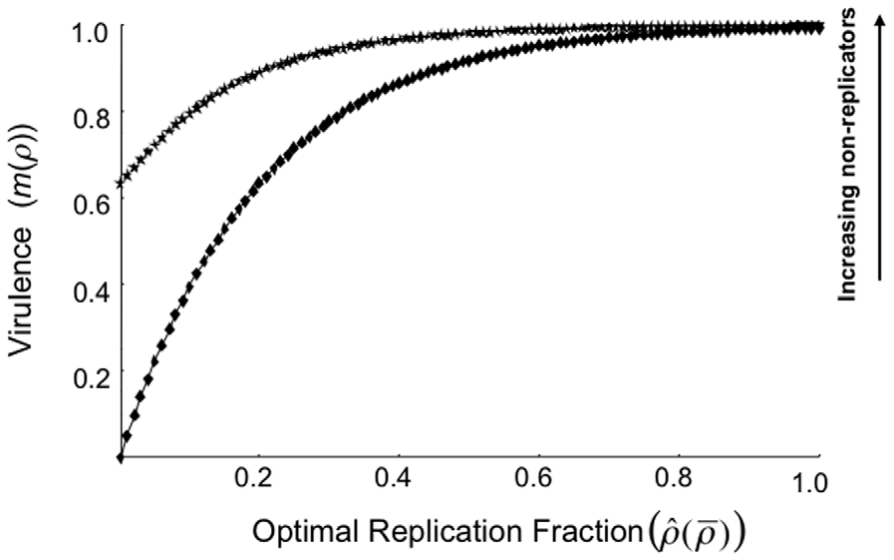
The role of non-replicating parasites on virulence (*m*(*ρ*)). Increases in the optimal replication fraction lead to increases in virulence and the presence of non-replicators can enhance virulence effects even if overall parasite fitness is restricted.

As outlined, virulence increases as the pathogen replication rate increases but saturates at high level of replication. However, the presence of non-replicating pathogens has a further increased effect on virulence as the optimal replication fraction increases (**Figure 2**). Given this, it is important to separate the effects of non-replication from cheating and kin competition. Non-replicating parasites may still contribute to pathogen-induced morbidity or mortality within a host (and consequently affect virulence) even if the effects of kin competition may be severe and act to limit overall parasite fitness.

The consequences of non-replicating pathogens on the host-pathogen interaction can be explored further by considering the conditions that would allow a rare novel variant of the disease (*I_n_*, *V_n_*) to invade and spread. This occurs if the net population growth rate of the novel variant is greater than zero (that is *dI_n_*/*dt*>0 *and dV_n_*/*dt*>0). This invasion analysis approach (which is similar to a standard local stability analysis where the resident strategy is at equilibrium) is an appropriate measure of fitness [43], [44] when density-dependent processes operate. Fitness is evaluated by taking the determinant of:
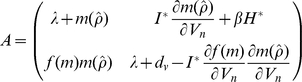
(12)and solving for the dominant eigenvalue, *λ* (where *I_n_* and *V_n_* are the infected hosts and free-living pathogen stages associated with the novel invading pathogen, respectively and H^*^ and I^*^ are the equilibrial abundances of susceptible hosts and hosts infected with the ancestral pathogen, respectively).

**Figure 3:**
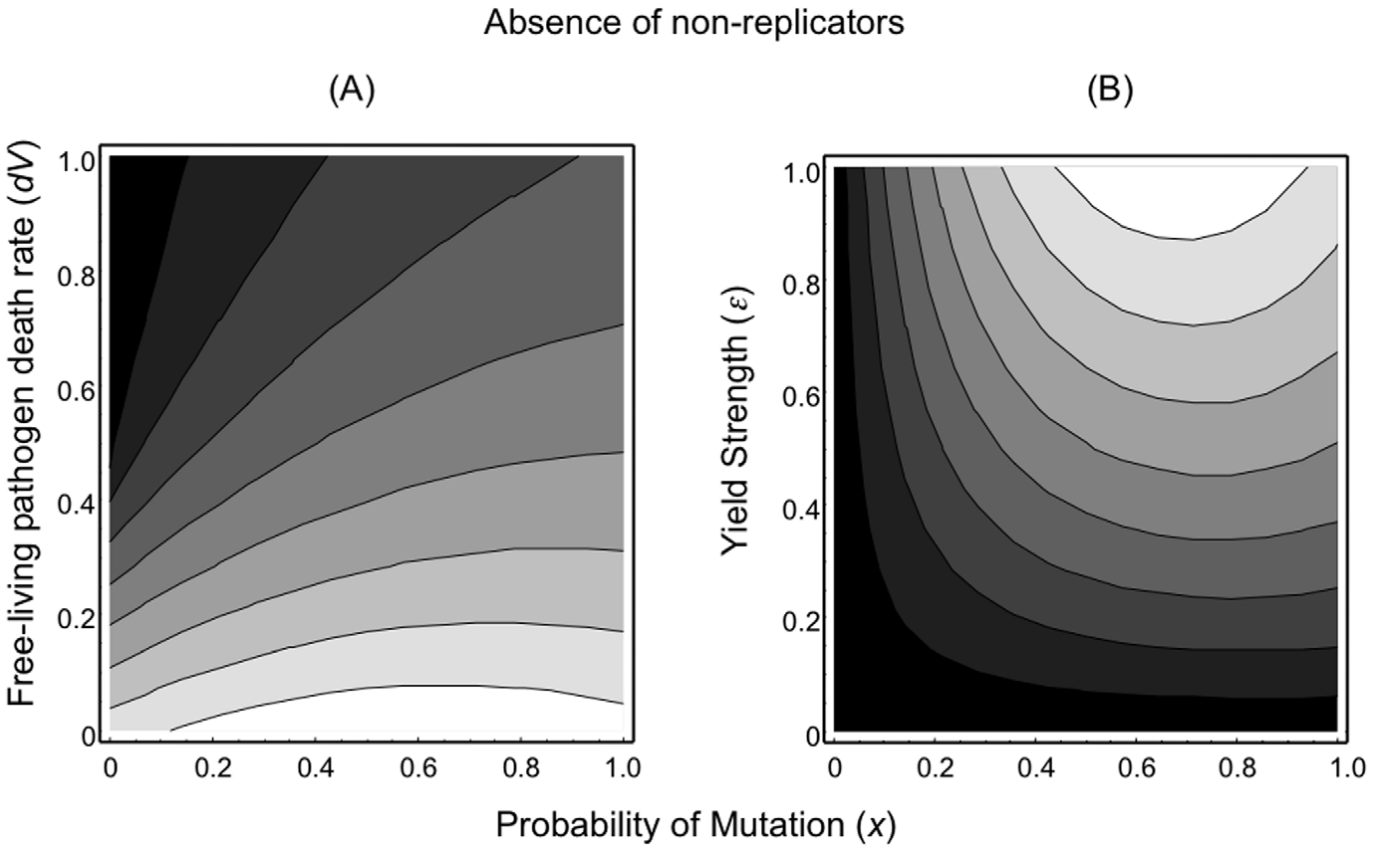
Fitness contours for rare disease variants in the absence of non-replicators. (A) Pathogen longevity (1/*d_V_*) – Mutation probability (*x*) and (B) Yield strength (*ε*) – Mutation probability (*x*). Long-lived pathogens and/or high yielding pathogens favoured the invasion of novel disease variants (those that have higher fitness). [Shading: black (low fitness) to white (high fitness)]. (Fitness is the dominant eigenvalue from equation 12).

In the absence of non-replicators, the invasion consequences of novel pathogens can be shown to depend on three main parasite life-history traits (**Figure 3**): free-living pathogen death rate (*d_V_*), yield strength (*ε*) and the mutation rate (a factor governing the degree of relatedness within a host – equation 8). Long-lived free-living pathogen stages (low *d_V_*) favour the invasion of novel disease (**Figure 3a**) particularly when parasites within a host are of intermediate relatedness or unrelated (∼0.6<*x*<1.0). Similarly, pathogens of intermediate to high relatedness together with high yields further increase the likelihood that a novel disease strategy can invade (**Figure 3b**).

**Figure 4:**
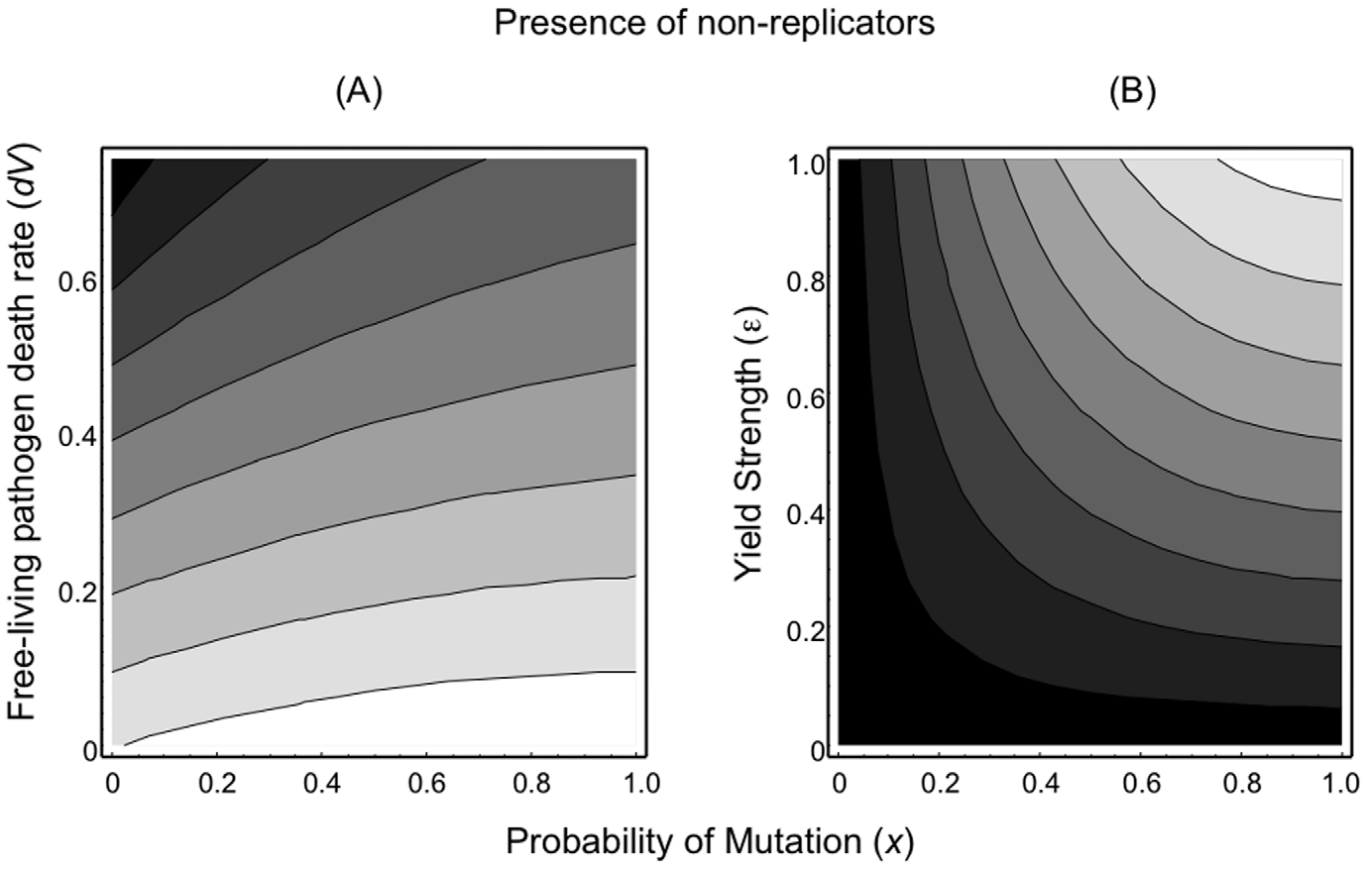
Fitness contours for rare disease variants in the presence of non-replicators. (A) Pathogen longevity (1/*d_V_*) – Mutation probability (*x*) and (B) Yield strength (*ε*) – Mutation probability (*x*). High levels of relatedness (parasite population structure) together with long-lived pathogens and/or high yielding pathogens favoured the invasion of novel disease variants (those that have higher fitness). [Shading: black (low fitness) to white (high fitness)]. (Fitness is the dominant eigenvalue from equation 12).

**Figure 5:**
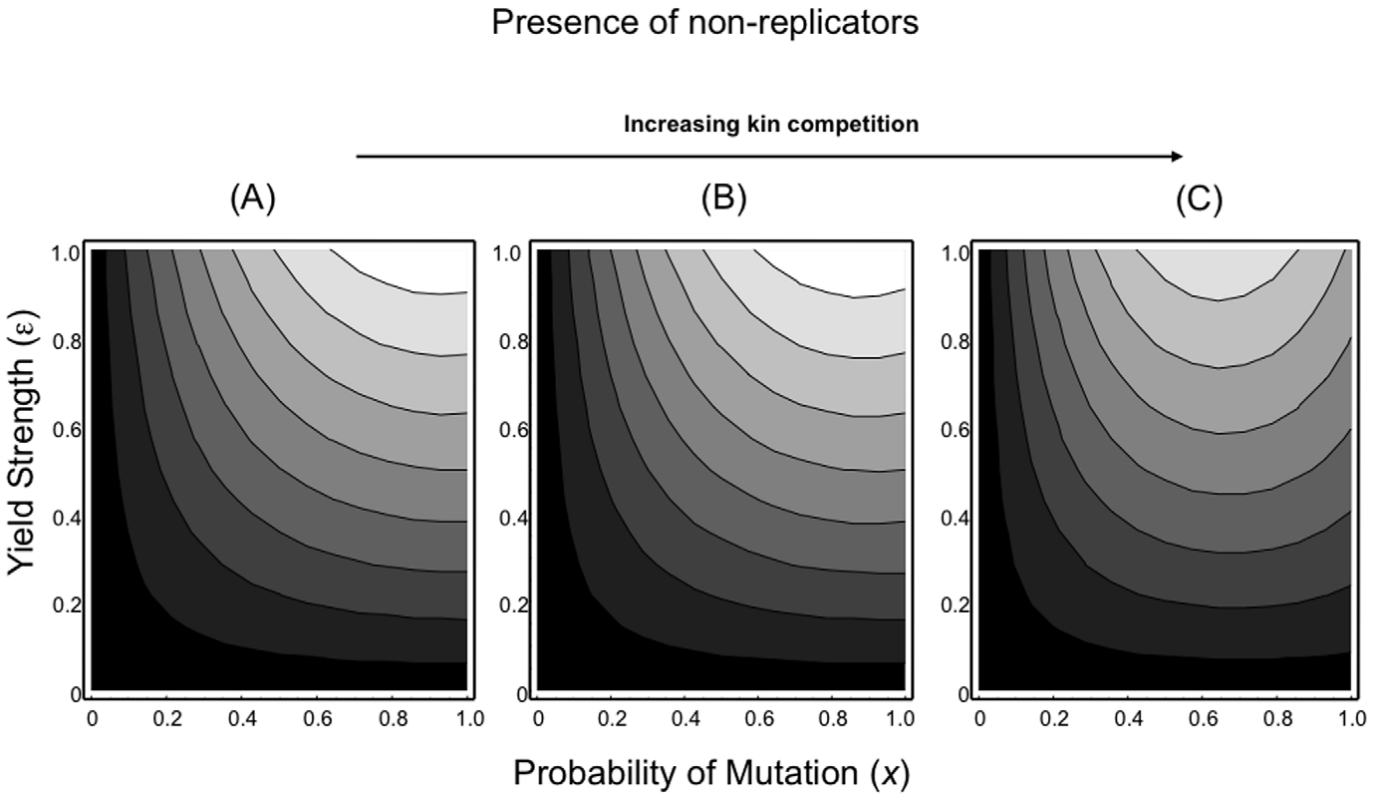
Fitness contours (Yield strength (*ε*) – Mutation probability (*x*)) for rare disease variants in the presence of non-replicators under varying degrees of kin competition. (A) α = 0.75, (B) α = 1.0, (C) α = 5.0. Increasing effects of kin competition can reduce optimal levels of relatedness and favour disease variants that produce fewer numbers of infectious pathogens (reduced yield). [Shading: black (low fitness) to white (high fitness)]. (Fitness is the dominant eigenvalue from equation 12).

In the presence of non-replicators (**Figure 4**), the previous patterns described hold: high yields and low death rates lead to pathogen invasion and spread. The presence of non-replicating parasites leads to a maximum in fitness when parasites are unrelated (*x*→1.0) (**Figure 4**). Alterations in the strength of kin competition (α) can also affect disease invasion potential (**Figure 5**). Increases in competition can offset the benefits to high yield and favour the invasion of pathogens that produce lower numbers of infectious particles. Furthermore, the degree of competition can also affect the optimal levels of relatedness within hosts. Strong competition within hosts leads to optimal yields at intermediate levels of relatedness. In contrast, reduced competition (cooperation) favours high yields at high levels of relatedness.

## Discussion

Here, the effects of how pathogen dynamics within-host scale up to affect dynamics between hosts have been explored. By considering the effects of parasite relatedness, it has been shown that the epidemiological outcome can depend on the degree of similarity between parasites in a host. In particular, increases in parasite relatedness led to a decline in the optimal replication fraction as the interaction between closely related individuals intensifies. Increases in competition can alleviate these interactions and contribute to parasite fitness.

The consequences of these within-host processes have important epidemiological consequences [5], [45] particularly in terms of parasite life history traits such as virulence. Several studies have highlighted that the effects of virulence can not be understood in isolation but must be considered within the context of host and parasite life histories [5], [10]–[11]. For instance, following death from infection, hosts release a yield of free-living pathogen, however this yield is constrained by the pathogen's speed of kill. Highly virulent parasites that kill hosts quickly yield fewer pathogens whereas more benign parasites may yield a higher number of transmissible agents. Selection will act differently depending on the interaction among factors such as the mode of transmission, speed of kill (virulence), free-living pathogen persistence time and within-host rate of replication.

Coupled with this is the fact that parasites within a host will be related (through the almost clonal expansion and proliferation on host resources) particularly if levels of recombination are low [28]. This will lead to competition between kin and non-kin and has consequences for virulence evolution and host-pathogen epidemiology. Here, it has been shown that increasing kin competition affects the invasion potential of pathogens such that maximum fitness is achieved under high pathogen yields and differing levels of relatedness. The presence of non-replicating parasites can create conditions where within-host levels of parasite relatedness are low even when kin competition is relatively weak (α<1). Increasing kin competition leads to an optimal invasion strategy similar to that observed when non-replicating parasites are absent.

Cooperation (and the potential for cheating) amongst parasites can take many forms – for instance it could involve participation in replication (and the production of shared toxins) to exploit hosts [46], [47], interference between non-kin through competition or collective action to avoid host defences. Cooperation between parasites increases the utilization of the host resource and can lead to a positive relationship between relatedness and virulence [23], [48]. Multiple infections are expected to reduce relatedness and have consequences for pathogen reproduction and virulence [47], [49]. However, the important issue here is not the relationship between virulence and relatedness but how individual parasites perform in individual hosts and how this translates to affect parasite life-history strategies. The presence of non-replicating (but otherwise equal) pathogens may still have positive fitness contributions to host exploitation and might act (sub)additively to increase virulence. The presence of non-replicators (and potentially non-kin) will alter the competitive environment and appropriate consideration of this alteration in the strength of competition is actually likely to be system-specific. Competition for limited resources even amongst closely related individuals will lead to winners and losers and within hosts this is likely to favour not only parasites that act to rapidly exploit hosts (and thereby the more virulent strains) but also virulence polymorphisms [14]. Strong competition increases parasite virulence as selection acts to promote more aggressive (resource-capturing) genotypes.

Virulence polymorphisms may arise through other manifestation of competition and host exploitation. Competition and exploitation of resources by pathogens will be different amongst hosts (due to host heterogeneity) and may lead to differential population and genetic structure between parasites [50]. As such, different levels of relatedness within hosts will lead to emergent phenomena such as virulence being localised and myopic [19]. Changes in the degree of relatedness will affect the optimal replication fraction and influence the observed level of virulence. As parasite virulence may be influenced by host and parasite life history characteristics [10]–[12] this also has important implications for host exploitation strategies [51]. High yielding and/or long-lived free-living infectious stages influence the evolutionary optimal strategy. This occurs through inclusive fitness benefits. Such parasite life histories have both direct effects on fitness (virulence-life history trait correlation) and indirect effects through changes in the levels of relatedness within hosts. Levels of selection may operate differently within and between hosts [52] to favour extended bouts of successful infections amongst hosts in a population [4].

Understanding the epidemiology of host-pathogen dynamics necessitates an appreciation of both the within-host [26], [27] and between host [37] dynamics. Understanding how selection operates on these various aspects of parasite fitness will reveal how parasites interact to affect epidemiological patterns [35]. Appreciating the finer implications of population and genetic structures will have important consequences not only for understanding pathogen evolutionary ecology but also for developing public health intervention programmes.
